# Supplementary material for: Intercropping Okra and Castor Bean Reduces Recruitment of Oriental Fruit Moth, Grapholita molesta (Lepidoptera: Tortricidae) in a Pear Orchard
Source: Insects. 2023 Nov 16;14(11):885. doi: 10.3390/insects14110885 (PMC10672554; doi:10.3390/insects14110885)
Supplement: Supplementary file 1 [file insects-14-00885-s001.zip › Table S1.pdf]

Table S1. Relative amounts of volatile compounds collected from okra leaves.

Compound	Rate time	CAS No.	Relative content (%)
2,2,3-trimethylpentane	10.874	564-02-3	21.57
Octamethylcyclotetrasiloxane	11.982	556-67-2	2.07
1,2-dichlorobenzene	13.456	95-50-1	1.56
Hexestrol	13.764	84-16-2	4.97
Unknown	14.489	-	6.13
1,4-diethylbenzene	16.686	105-05-5	trace
1-nonanal	17.357	124-19-6	1.49
2,4,6-trimethylbenzyl alcohol	18.055	4170-90-5	13.34
1-methyl-3-propan-2-ylbenzene	19.034	535-77-3	5.62
Thymol	19.285	89-83-8	11.67
α-copaene	19.638	3856-25-5	0.69
3-ethylbenzaldehyde	19.722	34246-54-3	4.21
Unknown	20.629	-	1.78
1-butan-2-yl-2,4-dimethylbenzene	23.015	1483-60-9	9.11
3,3-dimethyl-2-benzofuran-1-one	23.413	1689-09-4	7.74
1-o,2-o,3-o,5-o-tetrakis (trimethylsilyl)-D-xylofuranose	25.899	56271-68-2	trace
