# Supplementary material for: Intercropping Okra and Castor Bean Reduces Recruitment of Oriental Fruit Moth, Grapholita molesta (Lepidoptera: Tortricidae) in a Pear Orchard
Source: Insects. 2023 Nov 16;14(11):885. doi: 10.3390/insects14110885 (PMC10672554; doi:10.3390/insects14110885)
Supplement: Supplementary file 1 [file insects-14-00885-s001.zip › Table S2.pdf]

Table S2. Relative amounts of volatile compounds collected from okra flowers.

Compound	Rate time	CAS No.	Relative content (%)
Octamethylcyclotetrasiloxane	11.875	556-67-2	5.36
2,2,5,5-tetramethylhexane	11.976	1071-81-4	0.56
2-ethylbutyl methacrylate	13.317	5138-86-3	trace
1,2-dichlorobenzene	16.891	95-50-1	trace
Hexyl acetate	19.143	142-92-7	1.76
4-isopropylbenzyl alcohol	19.278	536-60-7	trace
Thymol	19.314	89-83-8	2.74
2-phenylethyl-1,1,2,2-d4-amine	19.667	87620-08-4	trace
6-methylhept-5-en-2-one	19.763	110-93-0	20.68
1,4-diacetylbenzene	19.839	1009-61-6	trace
Unknown	20.788	-	0.56
1-ethyl-3,5-dimethylbenzene	25.29	934-74-7	4.09
1,2-diethylbenzene	25.367	135-01-3	11.67
4-ethylacetophenone	25.552	937-30-4	9.81
Unknown	25.663	-	0.45
2-ethylhexanol	26.016	104-76-7	2.45
1,3,7-octatriene,3,7-dimethyl	26.359	502-99-8	7.28
1-ethyl-3-methyl-benzene	26.513	620-14-4	3.34
