# Supplementary material for: Intercropping Okra and Castor Bean Reduces Recruitment of Oriental Fruit Moth, Grapholita molesta (Lepidoptera: Tortricidae) in a Pear Orchard
Source: Insects. 2023 Nov 16;14(11):885. doi: 10.3390/insects14110885 (PMC10672554; doi:10.3390/insects14110885)
Supplement: Supplementary file 1 [file insects-14-00885-s001.zip › Table S3.pdf]

Table S3. Relative amounts of volatile compounds collected from okra fruits.

Compound	Rate time	CAS No.	Relative content (%)
2,2,3-trimethylpentane	12.057	564-02-3	16.57
1,2-dichlorobenzene	13.288	95-50-1	21.05
1,4-diethylbenzene	16.541	105-05-5	5.78
Decamethylcyclopentasiloxane	19.093	541-02-6	0.72
(3z)-3-hexen-1-yl acetate	19.538	3681-71-8	trace
4-lsopropylbenzyl alcohol	19.978	536-60-7	0.36
1-ethenyl-4-ethylbenzene	20.025	3454/7/7	1.45
Methyl jasmonate	21.233	39924-52-2	0.78
4-ethylacetophenone	23.041	937-30-4	3.19
Thymol	23.476	89-83-8	6.33
Unknown	24.047	-	trace
Unknown	25.577	-	13.18
1,4-diacetylbenzene	26.166	1009-61-6	0.67
3,3-dimethyl-2-benzofuran-1-one	26.443	1689-09-4	10.82
Unknown	27.241	-	trace
3,4-dimethylbenzoic acid TMS derivative	27.993	-	10.27
Tetradecamethyl cycloheptasiloxane	28.011	107-50-6	0.77
2,4,6-trimethylbenzyl alcohol	28.583	4170-90-5	trace
4-phenyl-2-butanol TMS derivative	28.912	-	4.81
Unknown	29.006	-	2.51
1-chlorooctadecane	30.437	3386-33-2	0.67
