# Supplementary material for: Intercropping Okra and Castor Bean Reduces Recruitment of Oriental Fruit Moth, Grapholita molesta (Lepidoptera: Tortricidae) in a Pear Orchard
Source: Insects. 2023 Nov 16;14(11):885. doi: 10.3390/insects14110885 (PMC10672554; doi:10.3390/insects14110885)
Supplement: Supplementary file 1 [file insects-14-00885-s001.zip › Table S4.pdf]

Table S4. Relative amounts of volatile compounds collected from castor bean leaves.

Compound	Rate time	CAS No.	Relative content (%)
2,2,3-trimethylpentane	11.988	564-02-3	0.67
Leaf alcohol	12.034	928-96-1	2.89
1,2-dichlorobenzene	12.537	95-50-1	6.81
Phenylacetaldehyde	13.229	122-78-1	19.01
1,4-diethylbenzene	16.543	105-05-5	0.38
Decamethylcyclopentasiloxane	19.088	541-02-6	trace
4-isopropylbenzyl alcohol	19.257	536-60-7	1.55
Dimethyl adipate	19.356	627-93-0	6.98
4-ethylacetophenone	19.615	937-30-4	trace
Unknown	20.017	-	trace
Dodecamethylcyclohexasiloxane	21.067	540-97-6	trace
Unknown	23.036	-	0.19
3,3-dimethyl-2-benzofuran-1-one	23.517	1689-09-4	4.88
Dibutyl phthalate	24.766	84-74-2	30.69
3,4-dimethylbenzoic acid TBDMS derivative	25.897	-	trace
2,6-Dimethylundecane	25.997	17301-23-4	0.69
Tetradecamethyl cycloheptasiloxane	26.145	107-50-6	trace
Hexadecamethylcyclooctasiloxan	26.308	556-68-3	5.78
Heptadecane	26.577	629-78-7	2.88
Unknown	27.907	-	0.79
Unknown	28.009	-	4.91
Unknown	28.481	-	0.11
4,6-dimethyl-dodecane	29.015	-	trace
Icosane	29.661	112-95-8	0.76
1-chlorooctadecane	29.786	3386-33-2	1.08
1-O-butyl 2-O-(8-methylnonyl) benzene-1,2-dicarboxylate	31.044	42343-36-2	7.17
