# Supplementary material for: Intercropping Okra and Castor Bean Reduces Recruitment of Oriental Fruit Moth, Grapholita molesta (Lepidoptera: Tortricidae) in a Pear Orchard
Source: Insects. 2023 Nov 16;14(11):885. doi: 10.3390/insects14110885 (PMC10672554; doi:10.3390/insects14110885)
Supplement: Supplementary file 1 [file insects-14-00885-s001.zip › Table S5.pdf]

Table S5. Relative amounts of volatile compounds collected from castor bean flowers.

Compound	Rate time	CAS No.	Relative content (%)
2,2,3-trimethylpentane	12.001	564-02-3	10.50
Dibutyl phthalate	12.781	84-74-2	21.03
β-damascone	13.231	23726-91-2	19.61
Unknown	13.542	-	0.98
1,4-diethylbenzene	16.538	105-05-5	0.49
dec-1-yne	19.244	764-93-2	4.69
Butane-2,3-diol	19.377	513-85-9	2.51
4-phenyl-2-butanol TMS derivative	20.069	-	9.11
1-O,2-O,3-O,5-O-tetrakis (trimethylsilyl)-D-xylofuranose	21.166	56271-68-2	7.96
4-ethylacetophenone	25.524	937-30-4	trace
Ethyl myristate	25.899	124-06-1	1.61
Cinnamaldehyde	26.793	104-55-2	3.66
Unknown	27.877	-	1.03
2,6-dimethylundecane	27.987	17301-23-4	0.27
1-iodoicosane	28.015	34994-81-5	3.01
Decamethylcyclopentasiloxane	29.533	541-02-6	trace
Heptadecane	29.729	629-78-7	11.44
1-chlorooctadecane	30.436	3386-33-2	trace
